# DNA content analysis allows discrimination between *Trypanosoma cruzi* and *Trypanosoma rangeli*

**DOI:** 10.1371/journal.pone.0189907

**Published:** 2017-12-19

**Authors:** Lucila Langoni Naves, Marcos Vinícius da Silva, Emanuella Francisco Fajardo, Raíssa Bernardes da Silva, Fernanda Bernadelli De Vito, Virmondes Rodrigues, Eliane Lages-Silva, Luis Eduardo Ramírez, André Luiz Pedrosa

**Affiliations:** 1 Departamento de Bioquímica, Farmacologia e Fisiologia, Instituto de Ciências Biológicas e Naturais, Universidade Federal do Triângulo Mineiro, Uberaba, Brasil; 2 Departamento de Imunologia, Microbiologia e Parasitologia, Instituto de Ciências Biológicas e Naturais, Universidade Federal do Triângulo Mineiro, Uberaba, Brasil; 3 Departamento de Clínica Médica—Disciplina de Hematologia e Hemoterapia, Universidade Federal do Triângulo Mineiro, Uberaba, Brasil; Instituto Butantan, BRAZIL

## Abstract

*Trypanosoma cruzi*, a human protozoan parasite, is the causative agent of Chagas disease. Currently the species is divided into six taxonomic groups. The genome of the CL Brener clone has been estimated to be 106.4–110.7 Mb, and DNA content analyses revealed that it is a diploid hybrid clone. *Trypanosoma rangeli* is a hemoflagellate that has the same reservoirs and vectors as *T*. *cruzi*; however, it is non-pathogenic to vertebrate hosts. The haploid genome of *T*. *rangeli* was previously estimated to be 24 Mb. The parasitic strains of *T*. *rangeli* are divided into KP1(+) and KP1(−). Thus, the objective of this study was to investigate the DNA content in different strains of *T*. *cruzi* and *T*. *rangeli* by flow cytometry. All *T*. *cruzi* and *T*. *rangeli* strains yielded cell cycle profiles with clearly identifiable G1-0 (2n) and G2-M (4n) peaks. *T*. *cruzi* and *T*. *rangeli* genome sizes were estimated using the clone CL Brener and the *Leishmania major* CC1 as reference cell lines because their genome sequences have been previously determined. The DNA content of *T*. *cruzi* strains ranged from 87,41 to 108,16 Mb, and the DNA content of *T*. *rangeli* strains ranged from 63,25 Mb to 68,66 Mb. No differences in DNA content were observed between KP1(+) and KP1(−) *T*. *rangeli* strains. Cultures containing mixtures of the epimastigote forms of *T*. *cruzi* and *T*. *rangeli* strains resulted in cell cycle profiles with distinct G1 peaks for strains of each species. These results demonstrate that DNA content analysis by flow cytometry is a reliable technique for discrimination between *T*. *cruzi* and *T*. *rangeli* isolated from different hosts.

## Introduction

Members of the genus *Trypanosoma* are protozoan parasites found worldwide and are capable of infecting humans, domestic and wild animals, and insects. *Trypanosoma cruzi* is the causative agent of Chagas disease, a chronic and debilitating disease that affects approximately 8 million people, mainly in Latin America [1]. *Trypanosoma rangeli* is also a protozoan parasite, which occurs in sympatry with *T*. *cruzi*. Despite *T*. *rangeli* infecting humans, it is considered nonpathogenic to the vertebrate hosts. However, *T*. *rangeli* infection can elicit the production of antibodies that cross-react with *T*. *cruzi* antigens. This may lead to the misdiagnosis of Chagas disease leading to a socioepidemiological impact and has not been considered by health authorities [2, 3]. *T*. *cruzi* population is divided in six genetic groups, viz., TcI–TcVI [4]. *T*. *cruzi* is considered diploid, but some parasitic strains are aneuploids because of a variation in the number of chromosomal bands or distribution of genetic markers, as determined by microsatellite (MS) typing [5, 6]. Sequencing of the clone CL Brener of *T*. *cruzi* revealed a haploid genome estimated to be 55 Mb [7]. Moreover, flow cytometric analysis using the clone CL Brener as the reference cell line demonstrated a variation in the nuclear genome size between *T*. *cruzi* groups, ranging from 80.64 Mb to 153.58 Mb [6, 8].

*T*. *rangeli* also possesses a high intraspecific variability, and analysis of kinetoplast DNA (kDNA) allowed the determination of two main genetic lineages in the parasite, viz., KP1(+) and KP1(−) based on the presence or absence of KP1 minicircles in the parasitic kDNA [9]. The division of *T*. *rangeli* into two main groups has been confirmed by several techniques, including RAPD [10], molecular karyotype [11], and terminal restriction fragment analyses [12]. However, analysis of other genetic markers, such as mini-exon, SSU rDNA, and CatLlike genes, detected increased variability allowing the division of the taxon into five groups, viz., TrA-TrE [13]. Recently, the investigation of single nucleotide polymorphisms (SNPs) and MS typing revealed a subdivision of the KP1(−) group, making a total of three *T*. *rangeli* groups [14]. Compared to other trypanosomatids, *T*. *rangeli* has the smallest genome sequenced thus far, with its haploid complement estimated to be 24 Mb [3].

Considering the limitations of serological methods for differential diagnosis of infections caused by *T*. *cruzi* and *T*. *rangeli*, several molecular methods have been developed for the differential diagnostic between these parasitic strains. Souto and colleagues demonstrated that analysis of the divergent D7a domain of rDNA permits simultaneous identification of *T*. *cruzi* and *T*. *rangeli* [15]. Ferreira and colleagues performed a comparative genome sequence analysis to identify molecular markers, which can specifically identify and distinguish between *T*. *cruzi* and *T*. *rangeli* [16]. Furthermore, DNA sequencing analysis of KP1(+) and KP1(−) strains of *T*. *rangeli* revealed the occurrence of a high frequency of nucleotide substitutions, which were named group specific substitutions (GSP) [16].

Despite several attempts for developing techniques for differential discrimination between *T*. *cruzi* and *T*. *rangeli*, investigating the DNA content of the parasitic strains has been explored limitedly. Thus, in this study, we investigated the DNA content of *T*. *cruzi* and *T*. *rangeli* by flow cytometry and demonstrated this approach to be a reliable alternative for discrimination between these species.

## Materials and methods

### Parasitic stocks

Six strains of *T*. *rangeli* (P02, P07, P19, Cas4, SO29, SO48, and LDG), five strains of *T*. *cruzi* (RN1, JG, Hel, and 3663), and two *T*. *cruzi* clones (CL Brener and Dm28c) were used. Strains 3663 (COLPROT 608) and Dm28c (COLPROT 010) were kindly provided by Dr. Claudia M d’Avila-Levy (Oswaldo Cruz Foundation, Rio de Janeiro, Brazil). *Leishmania major* CC1 clonal lineage was gently provided by Dr. Angela Kaysel Cruz (Faculdade de Medicina de Ribeirão–Universidade de São Paulo). *T*. *rangeli* strains, other *T*. *cruzi* strains were obtained from the laboratories of the disciplines of Parasitology and Immunology, Universidade Federal do Triângulo Mineiro, Minas Gerais, Brazil and previously described [12, 17].

### Culture conditions

Epimastigote forms of *T*. *cruzi* and *T*. *rangeli* were cultured in liver infusion tryptose (LIT) medium [18] supplemented with 10% fetal bovine serum. *T*. *rangeli* strains were cultured in LIT supplemented with 3% (v/v) human urine [19]. Promastigote forms of *L*. *major* were cultured in M199 supplemented as previously described [20].The cultures were incubated for four days at 28°C in a biochemical oxygen demand incubator (BOD).

### DNA extraction

DNA extraction of *T*. *cruzi* and *T*. *rangeli* epimastigote forms and *L*. *major* promastigote forms was performed with the Wizard^®^Genomic DNA Purification kit (Promega, Madison, Wisconsin, USA) according to the manufacturer’s instructions. Approximately 1 × 10^7^ parasite forms obtained from the exponential phase of growth curves were used for DNA extraction.

### Nucleic acid analysis

Subtelomeric duplex PCR was performed to exclude the possibility of cross-contamination in *T*. *cruzi* and *T*. *rangeli* cultures [21]. The final reaction mixture was 30 μL, containing 2.5 mM MgCl_2_, 0.24 mM dNTPs, 1 unit Taq DNA polymerase (Invitrogen), 30 mM KCl, 0.4 μM primers, and 50 ng genomic DNA. The primers used for *T*. *cruzi* detection were Tc189-fwd 5′-CCAACGCTCCGGGAAAAC-3′ and 189Rv3 5′-CGCTCTTCTCAGTATGGACTT-3′ and primers for detection for *T*. *rangeli* were TrF3 5′-CCCCATACAAAACACCCTT-3′ and TrR8 5′-TGGAATGACGGTGCGGCGAC-3′. PCR conditions were as follows: 94°C for 4 min; 35 cycles of 94°C for 40 s; 94°C and 65°C for 40 s; 72°C for 40 s; and final extension at 72°C for 10 min. The amplified products were observed on 1.5% agarose gel stained with ethidium bromide.

Genetic characterization of *T*. *rangeli* strains [9] was performed by multiplex PCR with the primers S35 5′-AAATAATGTACGGGTGGAGATGCATGA-3′, S36 5′-GGGTTCGATTGGGGTTGGTGT-3′, and KP1L 5′-ATACAACACTCTCTATATCAGG-3′ designed for KP1 minicircles in *T*. *rangeli*. KP1(+) strains yield a 165-bp fragment, a 300–450-bp band complex, and a 760-bp fragment. KP1(−) strains present all fragments except for the 165-bp fragment.

Genetic characterization of the *T*. *cruzi* strains was performed by PCR–restriction fragment length polymorphism (PCR-RFLP) using the TcSC5D (Sterol C-5 Desaturase from *T*. *cruzi*) gene as a target [22]. The primers TcSC5D-fwd (5′-GGACGTGGCGTTTGATTTAT-3′) and TcSC5D-rev (5′-TCCCATCTTCTTCGTTGACT-3′) amplify an 832-bp fragment that contains polymorphisms associated with restriction sites for endonucleases *Hpa*I and/or *Sph*I. In this fragment, *Hpa*I sites are found in homozygosity in TcI (generating fragments of 177 and 655bp) and TcII (231 and 601bp) strains and *Sph*I sites are also found in homozygosity in TcIII (337 and 495bp) strains. TcIV has no restriction sites for *Hpa*I or *Sph*I in the 832-bp fragment. *Hpa*I and *Sph*I sites are found in heterozygosity in the 832-bp fragments of TcV and TcVI (231, 337, 495, and 601bp) strains [22]. In order to discriminate between TcV and TcVI, we used primers Tc-Mec-kinase26-Fw (5′-TTTTTGCATGTCATTTTGG-3′) and Tc-Mec-kinase662-Rv (5′-AGCGGTCTTGTAATGAGCAC-3′) that amplify a fragment of 637bp *T*. *cruzi* mevalonate kinase (TcMK) gene [22]. *Xho*I digestion of the 637-bp fragment discriminates TcV (digestion) from TcVI (no digestion). The PCR protocols were conducted as described [22]. Briefly, the final volume of the reaction mixture was 25 μL, containing 10 pmol of each primer, PCR buffer (Invitrogen), 1.6 mM MgCl_2_, 50–100 ng genomic DNA, 200 mM dNTPs, and 1 unit Taq DNA polymerase (Invitrogen). PCR conditions were as follows: 94°C for 5 min; 35 cycles of 94°C for 30 s; 55°C for 30 s; 72°C for 30 s; and final extension at 72°C for 5 min for amplification of TcSC5D and at 94°C for 4.5 min, 35 cycles at 94°C for 30 s, followed by 30 s at 58°C, and 72°C for 30 s and final extension of 5 min at 72°C for amplification of TcMK [22]. The amplified products were observed on 1.2% agarose gel stained with ethidium bromide. Aliquots of 20 μL of the amplified products were digested with 1U of the enzyme *Hpa*I (NEB R105) at 55°C for 1 h and with 1U of the enzyme *Sph*I (NEB R0182) at 37°C for 1 h (TcSC5D fragment) or with one unit of *Xho*I (NEB R0146S) endonuclease (TcMK fragment). The resulting digestion fragments were observed on 1.5% agarose gel and stained with ethidium bromide.

### Flow cytometry analysis of *T*. *cruzi* and *T*. *rangeli*

To estimate the DNA content, aliquots with approximately 1 × 10^7^ epimastigote forms of *T*. *cruzi* and *T*. *rangeli* strains were centrifuged at 2000 ×g for 5 min at 4°C. The supernatant was discarded, and the cells were washed with ice cold 1× PBS. The pellet was suspended in 500 μL 1× PBS and 4.5 mL of 70% ethanol for fixation. The fixed cells were maintained at 4°C until flow cytometry DNA content analysis.

Propidium iodide (PI) staining was performed according to the method previously described [6]. Fixed cells were centrifuged at 2000 ×g for 5 min at 4°C. The supernatant was discarded, and the cells washed once with ice cold 1× PBS. The pellet was resuspended in 1 mL of a solution containing PI (100 μg/mL), Triton X-100 [0.1% (v/v)], and DNase free RNase A (200 μg/mL) and incubated for 15 min at 37°C in dark. Data acquisition was performed in a BD FACSCanto II Flow Cytometer (Becton, Dickinson and Company, USA). A minimum of 50,000 events were counted for each sample, at least in duplicates. Data analysis was performed using the FlowJo software (Tree Star Inc., Oregon, USA) and expressed as PI mean fluorescence intensity (MFI) in G0/G1 (2n) using FlowJo Cell Cycle identification tool. In brief, the parasitic strains were identified by the forward scatter (FSC) and side scatter (SSC) parameters, and debris and aggregates were excluded by using pulse area vs. pulse width and pulse area vs. pulse height.

Finally, PI fluorescence at 617 nm was evaluated by histograms. *T*. *cruzi* clone CL Brener was used as the reference for DNA content determination of all other parasitic strains analyzed because its genome size is known (106.4–110.7 Mb) [7] and its DNA content has been previously profiled by flow cytometry [6]. We also use the *L*. *major* CC1 clone as a reference because genome sequencing of three *L*. *major* strains revealed little variation in genome size [23]. In our analysis, we considered a value of 65.6Mb for a *L*. *major* diploid cell.

From fluorescence values at G1 peaks and genome sizes of reference strains, we determined linear regression curves that were used to estimate genomes of other *T*. *cruzi* and *T*. *rangeli* strains from their μ values. Furthermore, for estimating the genome sizes of *T*. *cruzi* and *T*. *rangeli* strains, the amount of *T*. *rangeli* kDNA was considered to be equivalent to the amount of *T*. *cruzi* kDNA [6, 8, 24]. For the statistical analysis of estimated genome size data (EGS), the Levene’s and Welch’s tests for homogeneity evaluation and the Komolgorov-Smirnov’s and Shapiro-Wilk's W tests were used to evaluate the normality of the distribution. The tests assumed non-parametric distribution and, therefore, the independent samples Mann-Whitney U test was used. The tests which p-value was less than 0.05 were considered significant. All analyzes were performed with the IBM® SPSS® Statistics version 20.0 program.

## Results

### Genetic identification of *T*. *cruzi* and *T*. *rangeli* strains

**Fig 1:**
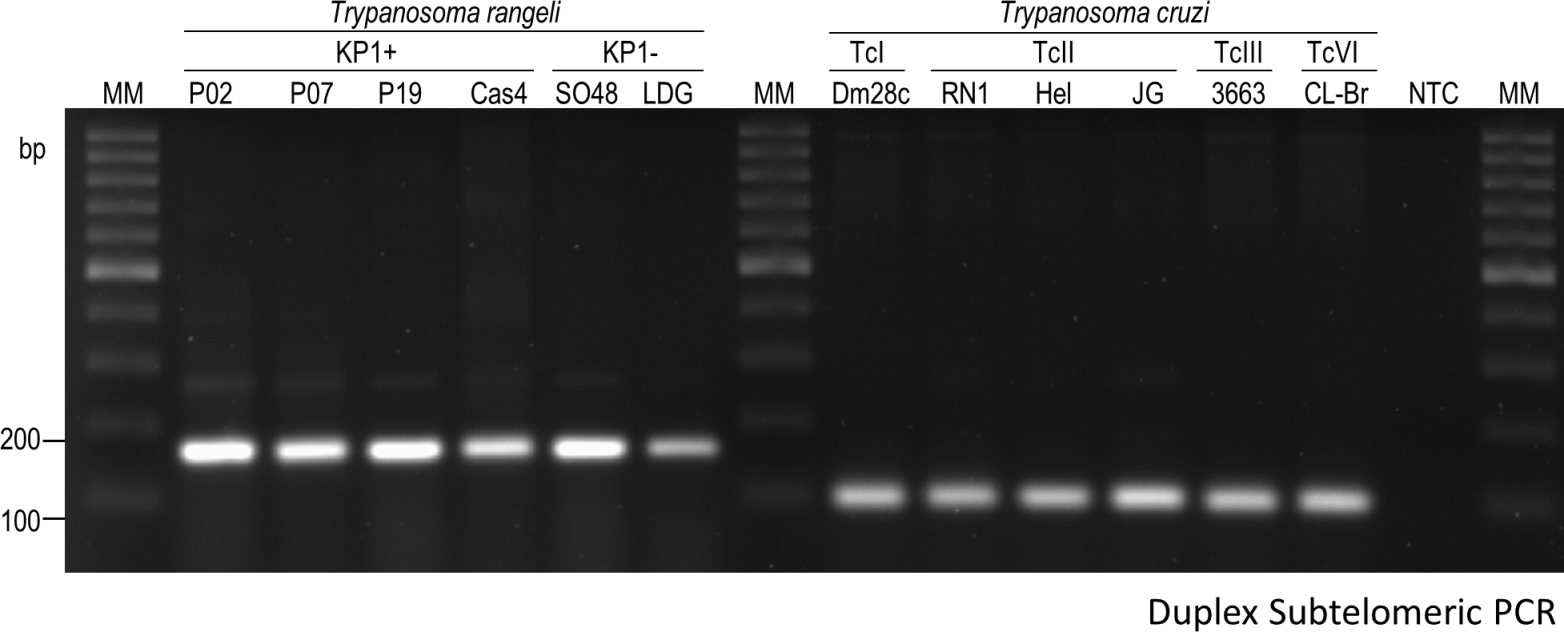
Genetic identification *Trypanosoma cruzi* and *Trypanosoma rangeli* strains. Duplex PCR to detect specific subtelomeric sequences in *T*. *cruzi* and *T*. *rangeli*. The amplified products were observed in 2.0% agarose gel stained with ethidium bromide. *Trypanosoma rangeli* strains: P02; P07; P19; Cas4; SO48; LDG. *T*. *cruzi* strains and clone: Dm28c; JG; RN1; Hel; 3663; CLBr (CL Brener). MM: Molecular marker 100bp. NTC: No-template control.

In order to ensure absence of cross-contamination in *T*. *cruzi* and *T*. *rangeli* cultures, we used a duplex-PCR protocol that allows the amplification of a fragment of 100bp from *T*. *cruzi* and a fragment of 170bp from *T*. *rangeli*. Accordingly, a 170-bp fragment was detected from all strains of *T*. *rangeli* and a 100-bp fragment was detected from all *T*. *cruzi* strains (Fig 1). Therefore, we conclude that there is no contamination of *T*. *rangeli* cultures with *T*. *cruzi* and vice-versa.

### Genetic characterization of *T*. *cruzi* and *T*. *rangeli* strains

**Fig 2:**
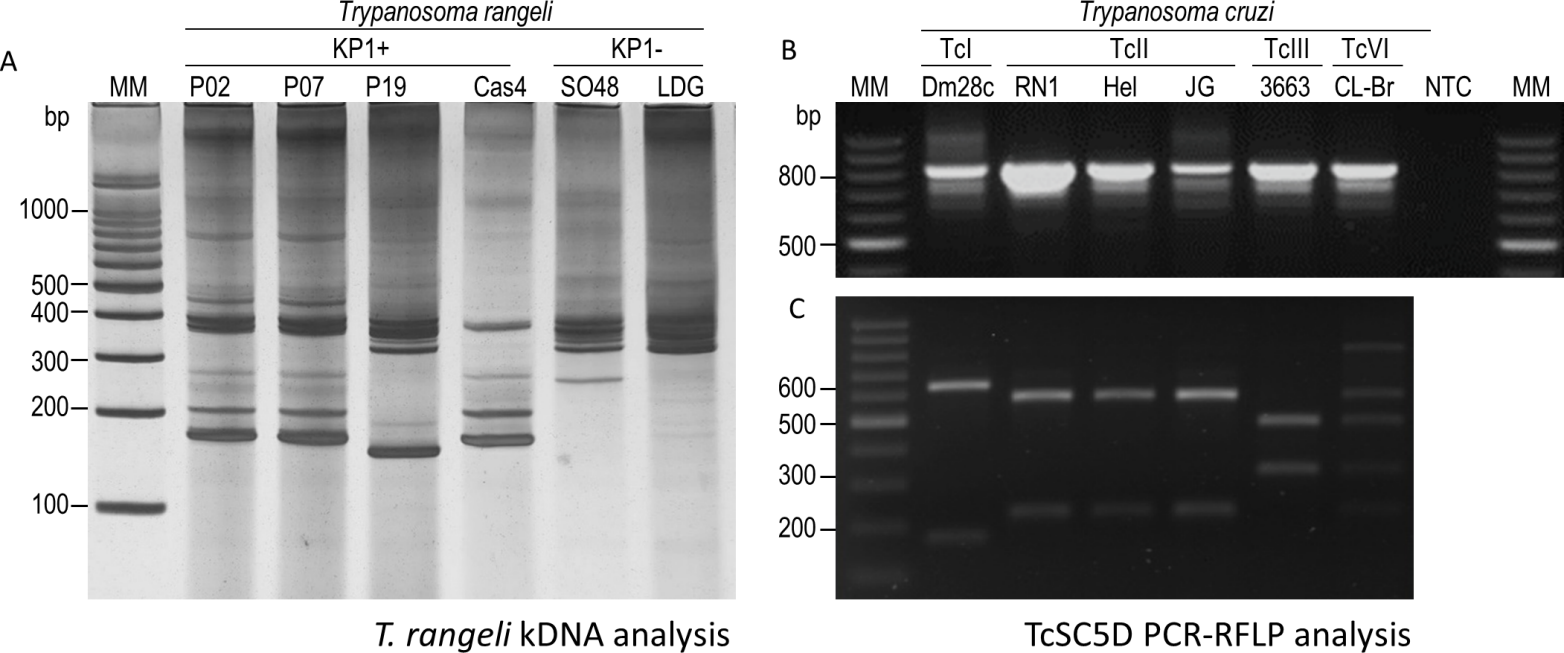
Genetic characterization *Trypanosoma cruzi* and *Trypanosoma rangeli* strains. (A) kDNA analysis of *T*. *rangeli* strains in a silver stained 6% polyacrylamide gel containing PCR products obtained with primers S35/S36/KP1L. The presence of a 165-bp band indicates the presence of KP1 minicircles. (B) Detection of the 832-bp fragment of TcSC5D gene in *T*. *cruzi* strains and clone. (C) PCR–RFLP of TcSC5D products digested with *Hpa*I and *Sph*I enzymes. MM: Molecular marker 100bp. NTC: No-template control.

Next, we characterize the main genetic groups of parasitic strains studied. Primers S35, S36, and KP1L were used to characterize kDNA minicircles in *T*. *rangeli* strains. As expected, all *T*. *rangeli* samples allowed the amplification of fragments between 300–400 bp (corresponding to KP3 minicircles) and a faint 760-bp fragment corresponding to the KP2 minicircles. Additionally, only P02, P07, P19, and Cas4 *T*. *rangeli* strains presented fragments in the size of approximately 165 bp, confirming their genotype as KP1(+) (Fig 2A).

PCR-RFLP of the TcSC5D gene was performed to determine the genetic groups of the *T*. *cruzi* strains. An 832-bp fragment was detected in all *T*. *cruzi* strains analyzed (Fig 2B). Next, each fragment was subjected to digestion with *Hpa*I and *Sph*I. Thus, strain Dm28c was classified as TcI (655 bp and 177 bp bands); strains RN1, JG, and Hel were classified as TcII (601 bp and 231 bp bands); strain 3663 was classified as TcIII (495 bp and 337 bp); and the results obtained for clone CL Brener (fragments of 601 bp, 495 bp, 337 bp, and 231 bp) are compatible with TcV or TcVI (Fig 2C). In order to confirm CL Brener genotype, we conducted a PCR-RFLP analysis of mevalonate kinase amplification product incubated with *Xho*I, determining its genotype as TcVI (S1 Fig).

**Fig 3:**
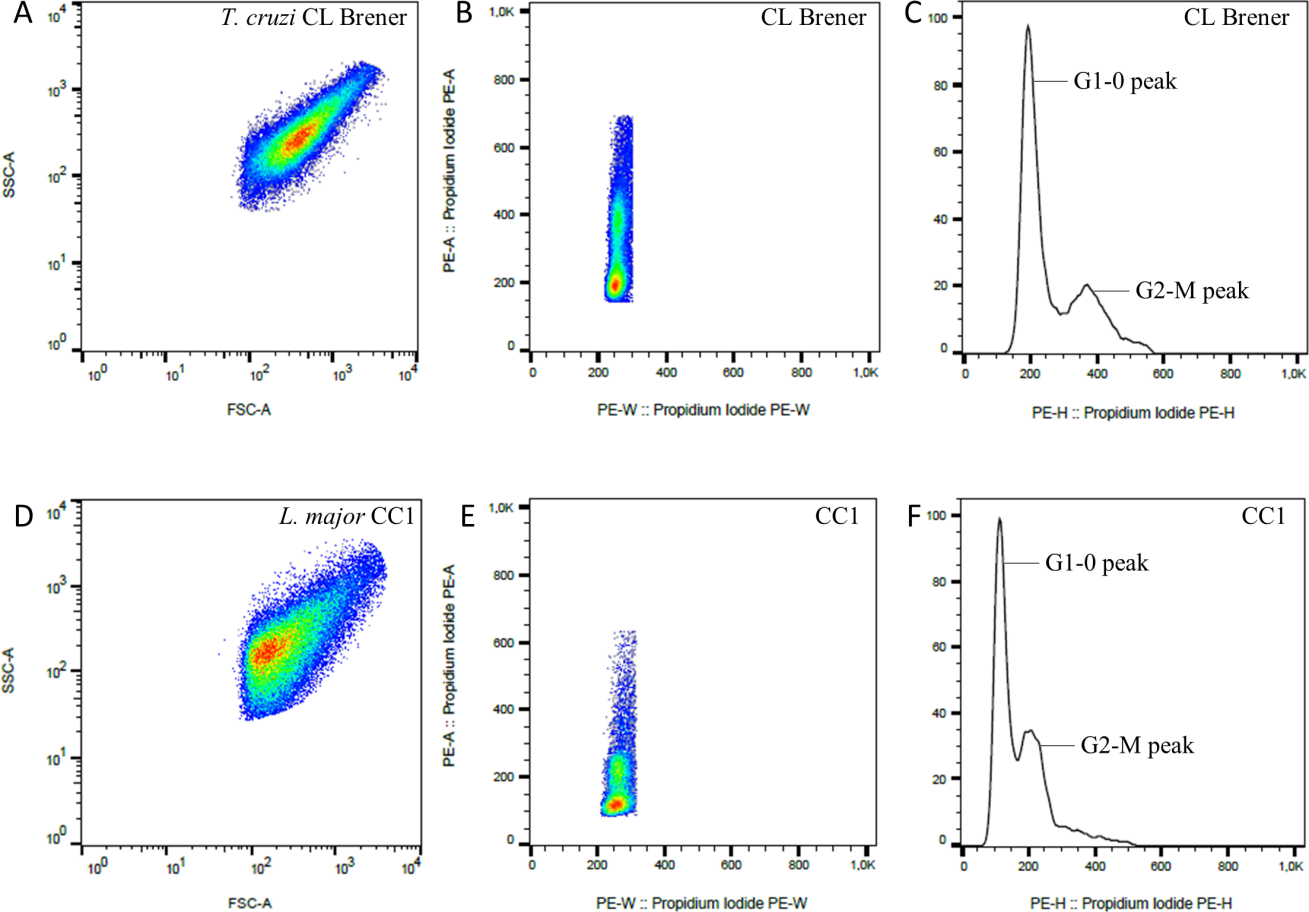
Representative flow cytometric gating strategy for DNA content analysis of *Trypanosoma cruzi* CL-Brener and *Leishmania major* CC1 strains stained with propidium iodide (PI). Panels A and D represent relative size (FSC) and complexity (SSC). Panels B and E panels represent PI-A and PI-W parameters indicating single cells stained with PI. Panels C and F show DNA content histogram and G1-0 and G2-M peaks are pointed. Relative PI fluorescence intensity at G1-0 was used for DNA content estimation for all strains analyzed using *T*.*cruzi* CL-Brener as reference strain.

Fig 3 represents the strategy used to analyze the DNA content of *T*. *cruzi* and *T*. *rangeli* strains using the *T*. *cruzi* CL Brener clone and the *L*. *major* strain CC1 as reference cell lines. In the parameter SSC-A and FSC-A (Fig 3A and 3D) it is possible to visualize the cellular populations in terms of complexity and size. Cells labeled with PI are shown in PI-A em PI-W parameters (Fig 3B and 3E). Fig 3C and 3F show histograms with distinct peaks in the different species showing cells in the G1-0 and G2-M phase of the cell cycle.

### Flow cytometry analysis of epimastigote forms of *T*. *cruzi* and *T*. *rangeli*

**Fig 4:**
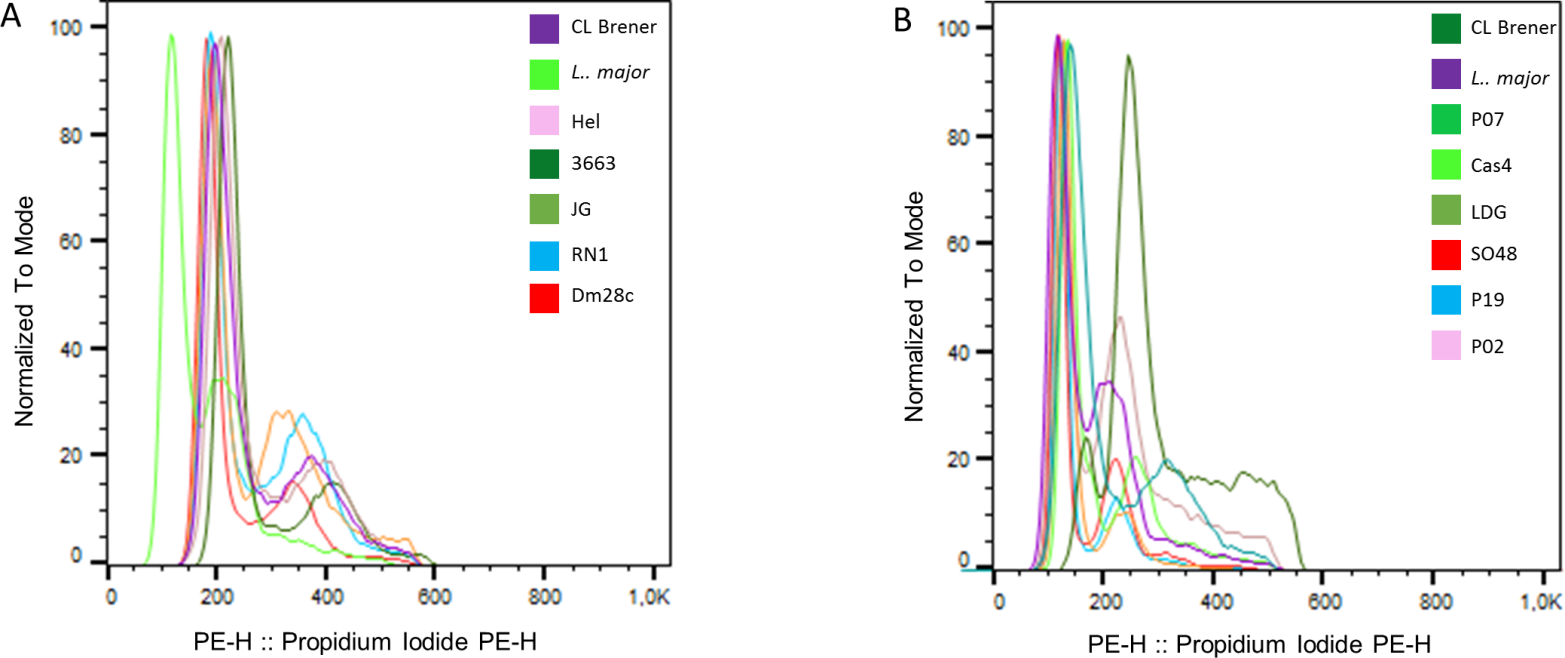
Flow cytometric analysis of the relative DNA content in *Trypanosoma cruzi* and *Trypanosoma rangeli*. (A) DNA histogram showing superimposed traces of the clone CL Brener, *T*. *cruzi* strains and *L*. *major*. (B) DNA histogram showing superimposed traces of the clone CL Brener, *T*. *rangeli* strains and *L*. *major*.

In this study, we included the clone CL Brener (TcVI) of *T*. *cruzi* and the CC1 strain of *L*. *major* as the reference cell lines in all DNA content analyses to estimate the genome size of the *T*. *cruzi* and *T*. *rangeli* strains studied. All *T*. *cruzi* and *T*. *rangeli* strains provided with clearly identifiable cell cycle profiles with G1-0 (2n) and G2-M (4n) peaks (Fig 4A and 4B).

**Fig 5:**
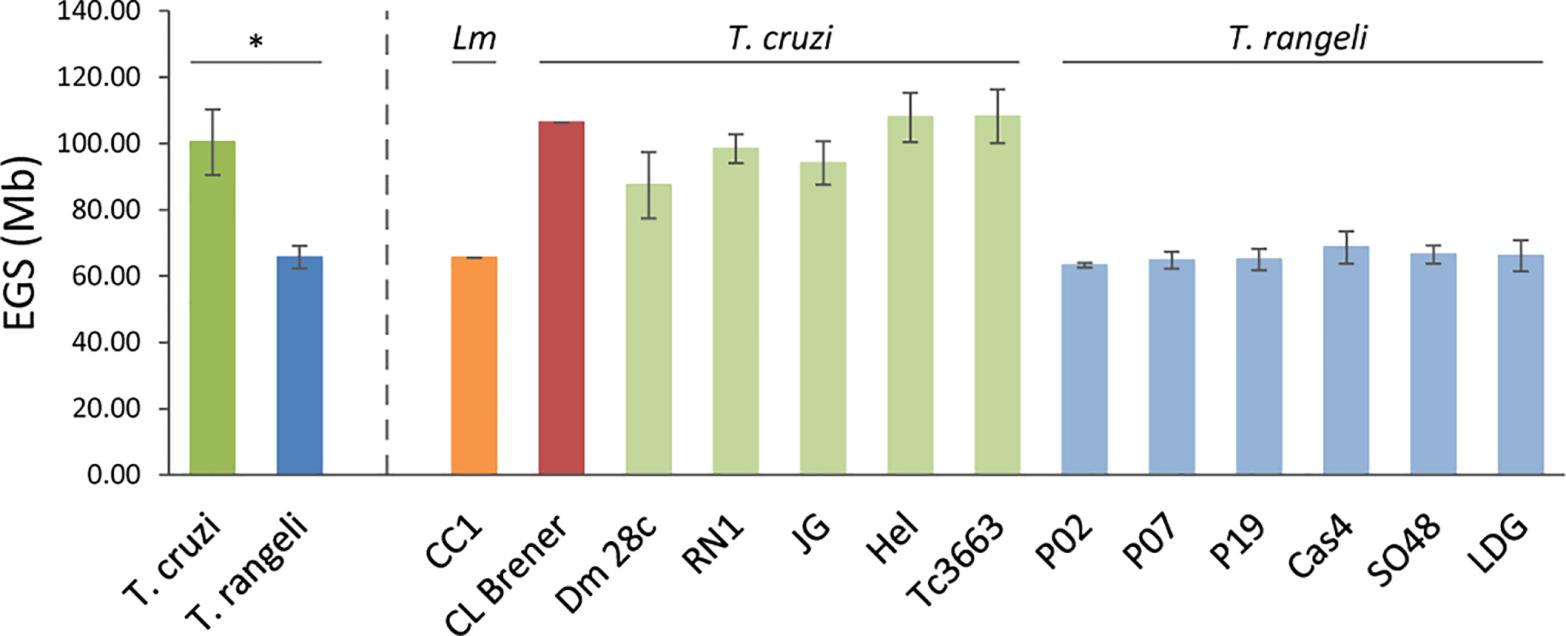
Determination of mean estimated genome sizes (EGS) of *Trypanosoma cruzi* and *Trypanosoma rangeli* strains and individual EGSs for each strain analyzed. On the left side of the figure the dark green and dark blue bars show the average size of the genomes of all strains of *T*. *cruzi* and *T*. *rangeli* species, respectively. The asterisk (*) indicates that there is a significant difference between the two groups (p<0.001). The orange bar represents the genome size *Leishmania major* (*Lm*) CC1 lineage (65,6Mb) and the red bar represents the genome size of *T*. *cruzi* CL Brener clone (106,4Mb)(both were used as reference cell lines for the estimation of the genomes sizes of the other strains). The light green bars represent the strains of T. cruzi (Dm28c, RN1, JG, Hel and 3663). The light blue bars represent *T*. *rangeli* strains (P02, P07, P19, Cas4, SO48 and LDG).

The ratio between these peaks (μ values) was in the range of 1.95–2.05, demonstrating the linearity between fluorescence intensity and DNA content of fixed parasitic strains. From FI determined for *T*. *cruzi* and *L*. *major* reference strains in three independent experiments, we determined equations of straight lines in order to estimate genome sizes of *T*. *cruzi* and *T*. *rangeli* strains (S2 and S3 Figs). Genome sizes of *T*. *cruzi* strains ranged from 87.41 Mb (Dm28c strain, TcI) to 108.16 Mb (3663 strain, TcIII). For *T*. *rangeli*, genomes sizes ranged from 63.25 Mb [P02 and strain, KP1(+)] to 68.66Mb [Cas 4 strain, KP1(+)] (Fig 5).

**Fig 6:**
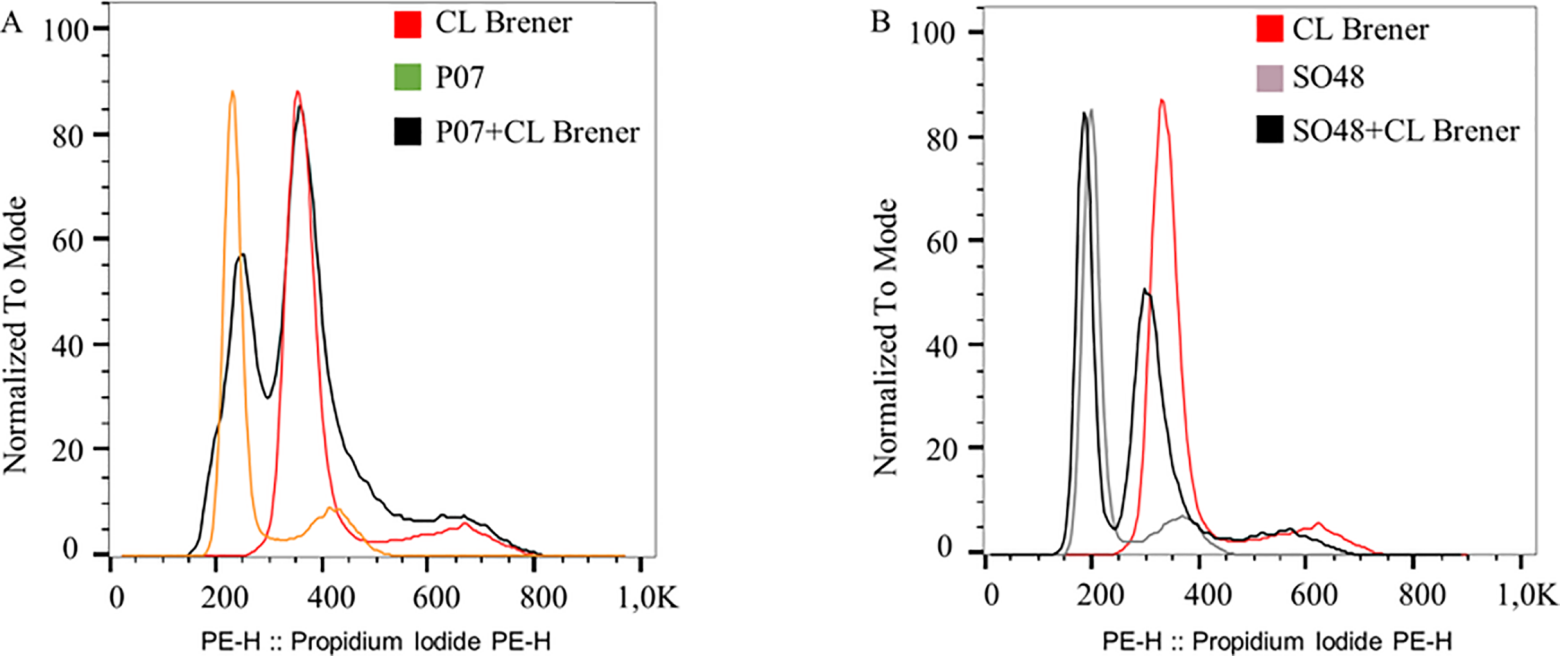
Discrimination between *T*. *cruzi* and *T*. *rangeli* by DNA content analysis. (A) DNA histogram of the clone CL Brener of *T*. *cruzi*, P07 strain (KP1+) of *T*. *rangeli*, and mixed CL Brener + P07. (B) DNA histogram of the clone CL Brener of *T*. *cruzi*, SO48 [KP1(−)] strain of *T*. *rangeli*, and mixed CL Brener + SO48.

The mean genome size for *T*. *cruzi* strains was 100.39±9.88 Mb, which was significantly higher than mean genome size of *T*. *rangeli* strains (65.71±3.37 Mb; p < 0.001). Mixtures of epimastigote forms of the clone CL-Brener of *T*. *cruzi* and *T*. *rangeli* strains resulted in histograms, which allowed discrimination between the G1 peaks of the two species (Fig 6A and 6B).

## Discussion

In this study, we have presented a novel strategy for discrimination between the main *Trypanosoma* spp. that infect humans in South America and Central America. We used PI-stained culture forms of two reference strains of trypanosomatids, *T*. *cruzi* CL Brener clone and *L*. *major* CC1 strain, that had their nuclear genome size determined by DNA sequencing [7, 23]. From these two points, we determine estimate genome sizes of *T*. *rangeli* and other *T*. *cruzi* strains.

The duplex-PCR subtelomeric protocol [21] excluded the possibility of cross-contamination between *T*. *cruzi* and *T*. *rangeli* cultures, allowing the use of the parasite forms for DNA content analysis. Next, we confirmed the genetic characterization of the parasites. *T*. *rangeli* genetic variability has been studied by several techniques and, according to the molecular marker used, parasite populations can be divided from two to five groups [17]. In our study, we investigated the organization of *T*. *rangeli* KP minicircles, because parasites from each group are associated with distinct vector species and the results of KP1 classification were confirmed by several other molecular markers [11, 12, 17, 25]. Accordingly, we observed the fragments amplified from the expected KP2 and KP3 kDNA minicircles in all *T*. *rangeli* strains and detected the presence of KP1 minicircles in the previously characterized strains [17, 26].The variation observed in the intensity of the KP2 fragments is consistent with the results previously described for other *T*. *rangeli* strains [9]. In the same way, all *T*. *cruzi* strains were associated with specific groups after PCR-RFLP analysis of polymorphisms in the SC5D and/or MK genes [22].

We investigated the DNA content of *T*. *rangeli* by flow cytometry and conducted a comparative analysis with the DNA content of *T*. *cruzi*. Considering the characteristics of flow cytometry, such as efficiency of PI incorporation by DNA and fluorescence compensation [27], the use of at least one reference cell line is mandatory for comparative analysis and consequent estimation of the parasitic genome size.

DNA content analysis of *T*. *cruzi* strains revealed that the genome size ranged from 85.2 Mb (Dm28c strain, TcI) to 113.0 Mb (Hel strain, TcII). These values are within the range of genome sizes determined for *T*. *cruzi* strains in previous studies that demonstrated a significant variability in the parasitic DNA content [6, 8]. Additionally, the genome size of Dm28c was estimated to be 85.2 Mb, which is very similar to the value estimated for other Dm28c strain which had its absolute DNA content previously determined [8]. This result demonstrates the accuracy of our estimates.

DNA content analysis of *T*. *rangeli* strains revealed that the genome size was significantly smaller, varying in size 61.7 Mb [P02 and P07 strains, KP1(−)] to 69.2 Mb [Cas 4 strain, KP1(+)]. No studies have demonstrated DNA content analysis by flow cytometry in *T*. *rangeli*; however, DNA sequencing has been performed, and the haploid genome size has been estimated to be 24 Mb [3]. Considering the differences between genome size estimation by flow cytometry and DNA sequencing, underrepresented sequences may account for part of the differences found between these values. Other possibility is a significant variation in the kDNA content between the two species. We consider in our analyses that the amount of kDNA is constant between parasite species and strains, as previously proposed for *T*. *cruzi* estimates [6]. However, the actual contribution of kDNA and nuclear DNA to the parasite’s total DNA content will require further investigation.

*T*. *rangeli* strains belonging to both KP1(+) and KP1(−) groups were used for determination of DNA content analysis. Previous studies on molecular karyotyping of these strains demonstrated differences in both the number and size of chromosomal bands between KP1(+) and KP1(−) groups of *T*. *rangeli* [11]. However, DNA content analysis between the strains of each group did not reveal significant differences between the two genetic groups of *T*. *rangeli*. Furthermore, cultures containing mixtures of the epimastigote forms of *T*. *cruzi* and *T*. *rangeli* strains resulted in cell cycle profiles with distinct G1 peaks for strains of each species. Accordingly, DNA content analysis by flow cytometry may be useful for species discrimination and for ploidy investigation in these parasites.

Ferreira and colleagues found a high frequency (1/56) of nucleotide substitutions when analyzing two loci of strains representing the main genetic groups of the parasite. This high intraspecific frequency of nucleotide substitutions is uncommon and has been called GSP to distinguish it from SNP, which occurs at a lower frequency in other organisms. In addition, sequencing the clones of P07 and Cas4 strains revealed the occurrence of heterozygosity in two loci [16], which may indicate the occurrence of hybridization in this parasite. In *T*. *cruzi*, TcIII and TcIV may be considered as homozygous hybrids, whereas TcV and TcVI are considered as heterozygous hybrids [28, 29]. Furthermore, *T*. *cruzi* “natural hybrids” are diploids, whereas hybrid lineages obtained under experimental conditions possess DNA content that is compatible to that of tetraploid cells [6]. *T*. *rangeli* P07 and Cas4 strains, isolated from *Didelphis albiventris* and *Rhodnius prolixus*, respectively, have the DNA content of a diploid cell. Recent studies suggested that the population structure of both *T*. *cruzi* and *T*. *rangeli* is primarily clonal [14, 30]. However, the main reproduction mechanism of this parasite remains controversial. Taken together, these results demonstrate that DNA content analysis by flow cytometry is a useful and reliable technique for discrimination between *T*. *cruzi* and *T*. *rangeli* isolated from different sources and may be applied for parasite identification if used in association with other approaches, such as PCR analysis. Further studies may reveal the extension of heterozygous loci in *T*. *rangeli* and other genetic and biological aspects of this parasite.

## Supporting information

S1 Fig Genetic characterization *Trypanosoma cruzi* CL Brener clone. Genetic characterization of the *T*. *cruzi* mevalonate kinase (TcMK) gene of CL Brener clone. The 537-bp fragment of TcMK gene CL Brener clone was *Xho*I-digested and the fragment was analysed in an 1.2% agarose gel stained with ethidium bromide. No digestion was observed in the fragment. CL-Br: *T*. *cruzi* CL Brener clone. MM: Molecular marker.
(TIF)

S2 Fig Determination of estimated genomes sizes of *Trypanosoma cruzi* and *Trypanosoma rangeli* strains. Three independent experiments were conducted using *Leishmania major* CC1 clone and *T*. *cruzi* CL Brener as reference strains. From the two points in each experiment corresponding to each of the reference strain we determined linear regression curves to calculate estimated genome sizes (EGSs) of other *T*. *cruzi* and *T*. *rangeli* strains. Dot colors in the graphs represent the following: green, *T*. *cruzi* strains; blue, *T*. *rangeli* strains; red, reference *T*. *cruzi* CL Brener clone, orange, reference *L*. *major* CC1 clone.
(TIF)

S3 Fig Determination of mean genome size of *Trypanosoma cruzi* and *Trypanosoma rangeli* strains. Values of estimated genome sizes (EGS) were determined from four independent experiments and are presented as mean ±SD. Dot colors in the graphs represent the following: green, *T*. *cruzi* strains; blue, *T*. *rangeli* strains; red, reference *T*. *cruzi* CL Brener clone, orange, reference *L*. *major* CC1 clone. SD: Standard deviation. R2: Correlation coefficient.
(TIF)
