# Supplementary figures and images for: DNA content analysis allows discrimination between Trypanosoma cruzi and Trypanosoma rangeli
Source: PLoS One. 2017 Dec 19;12(12):e0189907. doi: 10.1371/journal.pone.0189907 (PMC5736184; doi:10.1371/journal.pone.0189907)

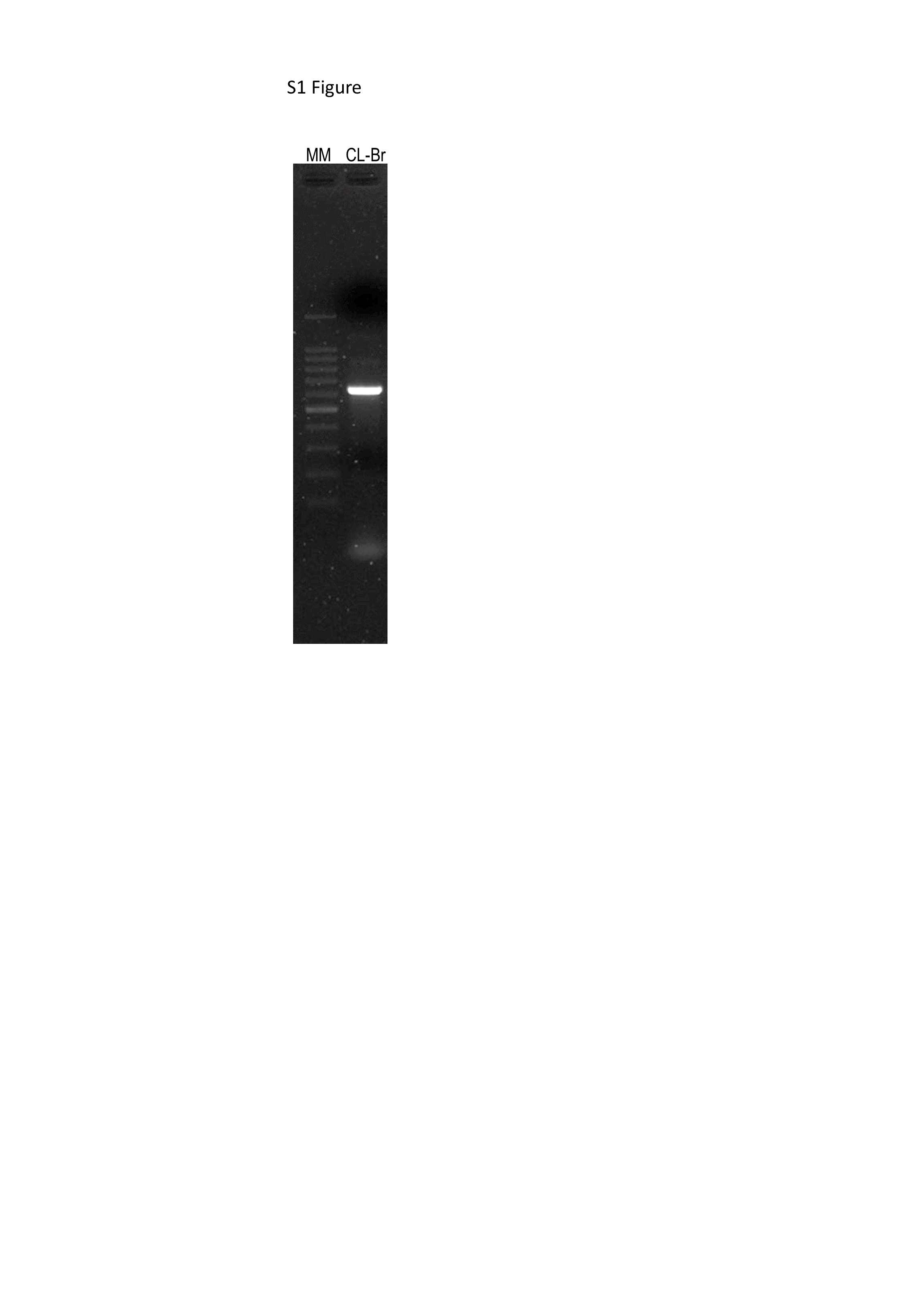

Supplement: S1 Fig — Genetic characterization of the T. cruzi mevalonate kinase (TcMK) gene of CL Brener clone. The 537-bp fragment of TcMK gene CL Brener clone was XhoI-digested and the fragment was analysed in an 1.2% agarose gel stained with ethidium bromide. No digestion was observed in the fragment. CL-Br: T. cruzi CL Brener clone. MM: Molecular marker. (TIF) [file pone.0189907.s001.tif]

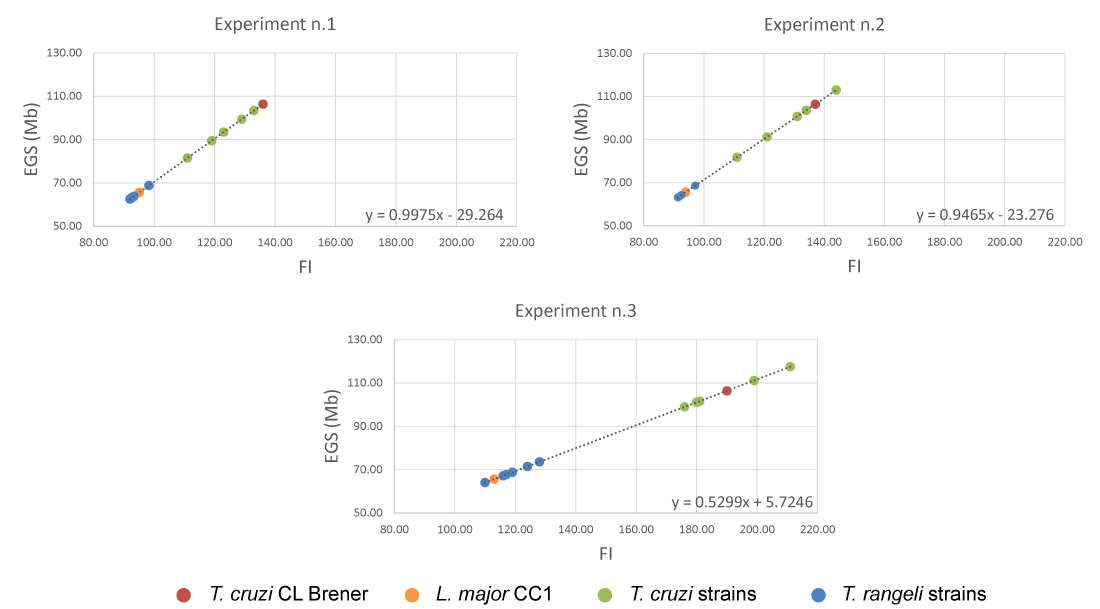

Supplement: S2 Fig — Three independent experiments were conducted using Leishmania major CC1 clone and T. cruzi CL Brener as reference strains. From the two points in each experiment corresponding to each of the reference strain we determined linear regression curves to calculate estimated genome sizes (EGSs) of other T. cruzi and T. rangeli strains. Dot colors in the graphs represent the following: green, T. cruzi strains; blue, T. rangeli strains; red, reference T. cruzi CL Brener clone, orange, reference L. major CC1 clone. (TIF) [file pone.0189907.s002.tif]

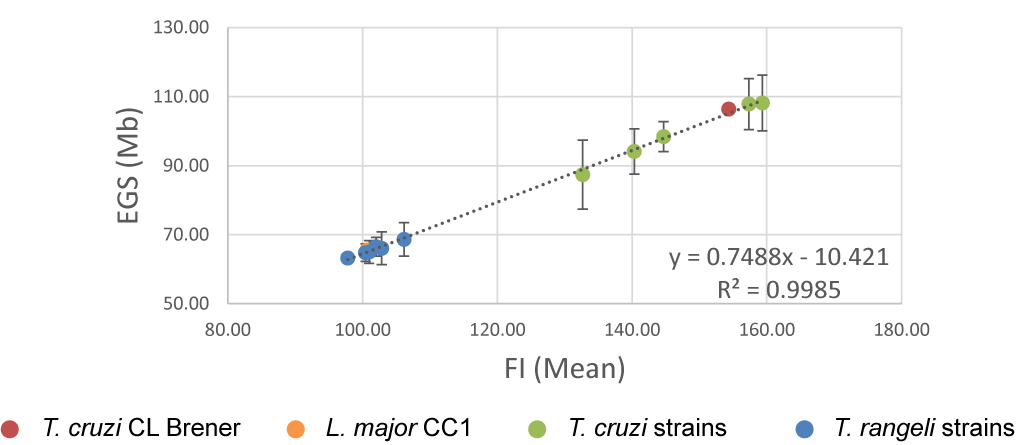

Supplement: S3 Fig — Values of estimated genome sizes (EGS) were determined from four independent experiments and are presented as mean ±SD. Dot colors in the graphs represent the following: green, T. cruzi strains; blue, T. rangeli strains; red, reference T. cruzi CL Brener clone, orange, reference L. major CC1 clone. SD: Standard deviation. R2: Correlation coefficient. (TIF) [file pone.0189907.s003.tif]
